# Integrated diagnosis and time-series sensitivity evaluation of nutrient deficiencies in medicinal plant (*Ligusticum chuanxiong* Hort.) based on UAV multispectral sensors

**DOI:** 10.3389/fpls.2022.1092610

**Published:** 2023-01-10

**Authors:** Wenbo Li, Ke Wang, Guiqi Han, Hai Wang, Ningbo Tan, Zhuyun Yan

**Affiliations:** ^1^ State Key Laboratory of Southwestern Chinese Medicine Resources, Chengdu University of Traditional Chinese Medicine, Chengdu, China; ^2^ School of Big Data and Artificial Intelligence, Chengdu Technological University, Chengdu, China

**Keywords:** nutrient deficiency, symptom identification, unmanned aerial vehicle (UAV), canopy reflectance, medicinal plants, *ligusticum chuanxiong* Hort

## Abstract

**Background:**

Nitrogen(N), phosphorus(P), and potassium(K) are essential elements that are highly deficient during plant growth. Existing diagnostic methods are not suitable for rapid diagnosis of large-scale planting areas. Near-ground remote sensing technology based on unmanned aerial vehicle (UAV) and sensor is often applied to crop growth condition monitoring and agricultural management. It has been proven to be used for monitoring plant N, P, and K content. However, its integrated diagnostic model has been less studied.

**Methods:**

In this study, we collected UAV multispectral images of Ligusticum chuanxiong Hort. in different periods of nutritional stress and constructed recognition models with different heights and algorithms. The optimal model variables were selected, and the effects of different sampling heights and modeling algorithms on the model efficiency under the time span were evaluated. At the same time, we evaluated the timeliness of the model based on leaf element content determination and SPAD. It was also validated in field crop production.

**Results:**

The results showed that the LR algorithm’s model had optimal performance at all periods and flight altitudes. The optimal accuracy of N-deficient plants identification reached 100%, P/K-deficient plants reached 92.4%, and normal plants reached 91.7%. The results of UAV multispectral diagnosis, chemical diagnosis, and SPAD value diagnosis were consistent in the diagnosis of N deficiency, and the diagnosis of P and K deficiency was slightly lagging behind that of chemical diagnosis.

**Conclusions:**

This research uses UAV remote sensing technology to establish an efficient, fast, and timely nutritional diagnosis method for L. Chuanxiong, which is applied in production. Meanwhile, the standardized production of medicinal plant resources provides new solutions.

## 1 Introduction

There are 14 essential mineral nutrients in the whole life cycle of plants (de Bang et al., 2021), among which nitrogen(N), phosphorus(P), and potassium(K) are closely related to the yield and quality of cultivated crops and are more likely to be deficient (Sanchez et al., 2020). N is a component of plant proteins, nucleic acids, chlorophyll, and other substances. N deficiency can cause phenotypic symptoms such as stunted growth, yellowing old leaves, small leaves, and reduced branching and flowering (Rahayu et al., 2005). P is an element involved in energy metabolism (ATP, NADPH), nucleic acids, and membrane phospholipid composition (Kamerlin et al., 2013). P deficiency causes a reduction in cell division and elongation, reddish-purple or dark green plant leaves, and stunted plant growth and development (Hughes and Lev-Yadun, 2015). K regulates plant growth in plants by affecting electroneutrality, osmoregulation, anion-cation balance, and biochemical pH status, and K+ reduces the production of reactive oxygen species (ROS) by suppressing the number of electrons used for side reactions with oxygen, such that potassium deficiency can lead to local necrosis of the plant foliage (Pottosin and Shabala, 2016). K deficiency also predisposes the plant to collapse by hindering cell wall development (Anschutz et al., 2014). Identifying and replenishing N, P, and K deficiencies at an early stage of plant deficiency is the key to ensuring proper plant growth. Therefore, N, P, and K are the plant nutrients that need to be monitored as a priority in field production management.

*Ligusticum chuanxiong* Hort. is one of the commonly used medicinal plants of the Umbelliferae family, which has been cultivated in China for more than 1500 years (Ran et al., 2011). Its roots are widely used in China, Japan, Korea, Singapore, and other Asian regions for treating and preventing cardiovascular and gynecological diseases (Chen et al., 2018). Currently, the cultivation area of L. chuanxiong in the Chengdu Plain of China is more than 6000 hm² year-round, with an annual production of 1.8×107~20×107 kg (Peng et al., 2020). However, irrational fertilization exists in the process of large-scale cultivation. This causes a waste of resources (Krasilnikov et al., 2022), environmental pollution and damages the quality of Chuanxiong herbs (Liu, 2009; Chen et al., 2022).On the other hand, due to the specificity of their use, medicinal plants are often subject to strict requirements in terms of growing environment and cultivation management, which requires a large amount of labor. With the urbanization and aging of China’s population, labor management costs have increased. Therefore, in the context of large-scale cultivation and rising labor costs, there is an urgent need for efficient and reliable tools to assist medicinal growers in management and decision-making.

In the process of crop planting and production, due to the differences in soil properties and nutrient content, as well as temperature changes, rainfall conditions, etc., the nutrient loss is different (St Luce et al., 2011). Adequate fertilization is an important factor to ensure crop yield and quality (Imran et al., 2021). Therefore, it is necessary to monitor the nutritional status of the key stages of crop growth to take timely remedial measures. At present, the nutritional diagnosis of crops mainly includes sensory empirical, chemical, and spectral. Sensory experience diagnosis is highly subjective. Chemical diagnosis relies on laboratory conditions, and the operation process is cumbersome and time-consuming (Daughtry et al., 2000). The spectral diagnosis method established by using the close correlation between crop nutritional status and its spectral characteristics is fast, non-destructive, and easy to grasp (Balasubramanian et al., 1998; Toth and Jozkow, 2016; Sanchez et al., 2020). Although the existing proximal spectral diagnosis technology identifies more types of element deficiencies with high accuracy (Rustioni et al., 2018; Sanchez et al., 2020), the collection efficiency is low and cannot meet the real-time monitoring of large-scale agricultural fields. And with the development of UAV technology, it is equipped with different sensors such as RGB, Multispectral, Hyperspectral, Thermal Sensor, Light Detection and Ranging (Sun et al., 2022). Appropriate sensors can be selected according to the application (Zhu et al., 2021), thus providing a new solution for crop growth monitoring (Toth and Jozkow, 2016). UAVs are equipped with optical sensors to collect and quantify light attenuation caused by photon scattering, absorption, and transmission caused by the interaction of light with plant canopy tissue. These interrelationships are closely related to the physical and chemical properties of the plant, thus obtaining crop phenotypic parameters to provide an accurate and timely assessment of the crop development status (Homolova et al., 2013), such as the assessment of crop nutrition, disease, pest incidence, weeds, biomass, etc. (Osco et al., 2020; de Castro et al., 2021; Rehman et al., 2022). At present, the acquisition of near-Earth spectral image technology based on UAV has attracted the attention of many scholars due to its high efficiency, real-time and non-destructive characteristics.

The sustainable development of agroecosystems needs to be considered in crop growth detection. Non-destructive, low-cost, and high-efficiency UAV multispectral technology solves the problem. Multispectral cameras have three or more discrete bands. The choice of bands depends on the need for vegetation indices (VI) associated with crop phenotypes, which are more sensitive to vegetation characteristics than a single wavelength. Among them, indices such as Normalized Difference VI (NDVI), Green Normalized Difference VI (GNDVI), Normalized Difference Red-edge Index (NDRE), and soil-adjusted VI (SAVI) are considered to be closely related to the nutritional status of plants (Osco et al., 2020). Rehman et al. (2022) used NDVI and NDRE to establish a prediction model for rice nitrogen and yield in different locations and time spans. Gordillo-Salinas et al. (2021) found that GNDVI and Blue Normalized Difference Vegetation Index (BNDVI) had better prediction effects on the nitrogen content of wheat in different phenological periods. Furlanetto et al. (2021) found that GNDVI, NDVI, Ratio between Infrared and Green (GRVI), Ratio between Green and Infrared (GNIR), Ratio between Red and Infrared (RNIR), and Ratio between Infrared and Red (RVI) can effectively differentiate adequate K supply maize plants under treatment with severe potassium deficiency. Gracia-Romero et al. (2017) found that the NDVI, SAVI, Renormalized difference vegetation index (RDVI), Enhanced vegetation index (EVI) and other indices of corn plants with and without phosphate fertilizer had significant changes. Given this, we believe that UAV multispectral technology has the potential for integrated diagnosis of plant N, P, and K deficiency and can meet the needs of future crop cultivation and production self-energy and intelligence.

This study aimed to verify the possibility of distinguishing N, P and K deficiency in plants using UAV multispectral technology. And we will evaluate the impact of different algorithms and flight altitudes on classification accuracy as well as the timing of the diagnosis compared to other diagnostic methods. We expect that UAV multispectral technology with a suitable algorithm and flight altitude can accurately identify deficient plants and can detect the deficiency symptoms of plants as early as possible.

## 2 Materials and methods

**Figure 1:**
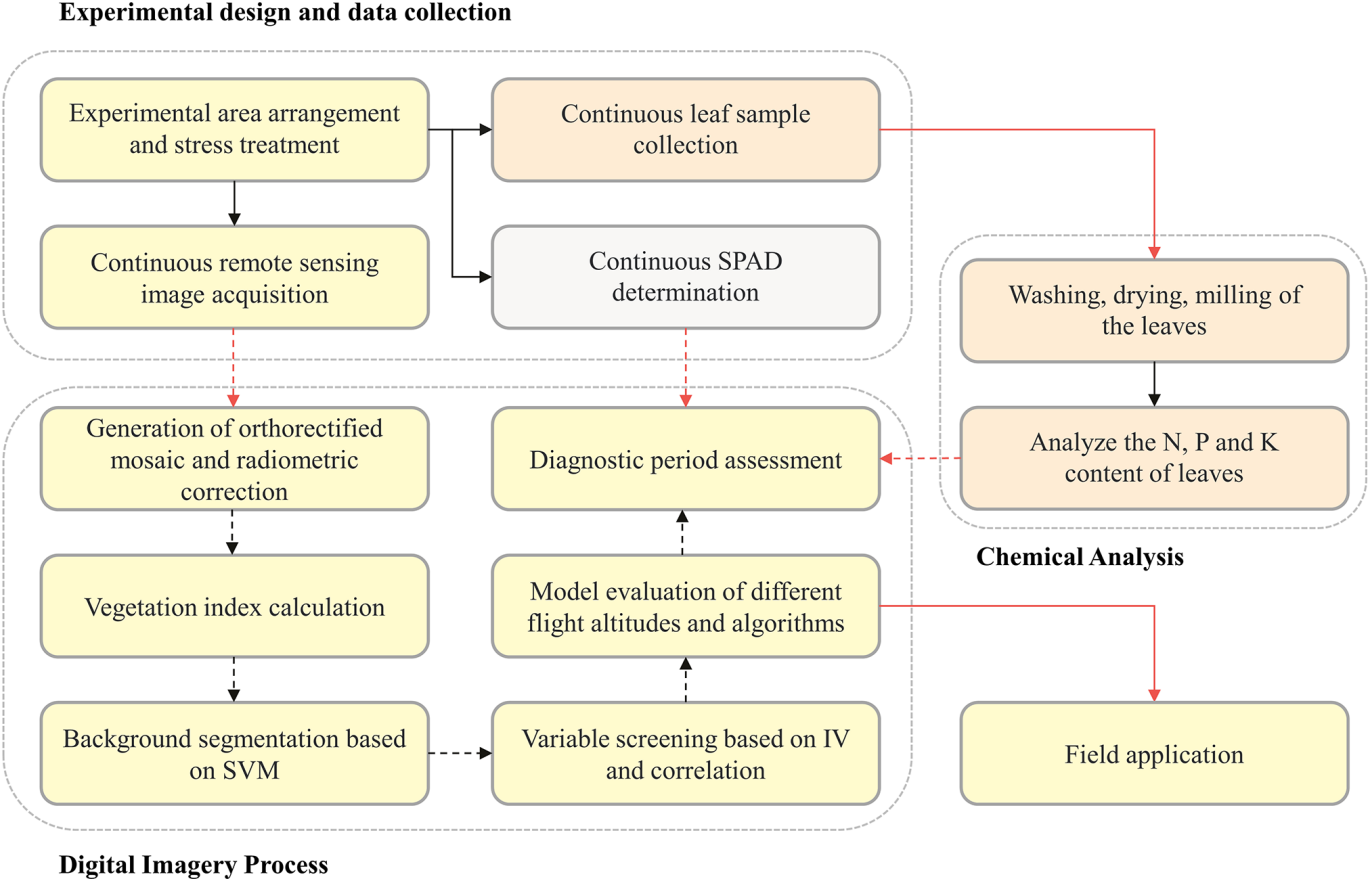
Workflow of the process performed in this study.

The method is described in three main stages: a) experimental design and data collection; b) digital image processing and data analysis; c) Chemical analysis of leaf tissue and determination of growth indicators. The specific steps of each phase are organized in a workflow (**Figure 1**) and detailed below.

### 2.1 Experimental design and data collection

#### 2.1.1 Study area and experimental design

**Figure 2:**
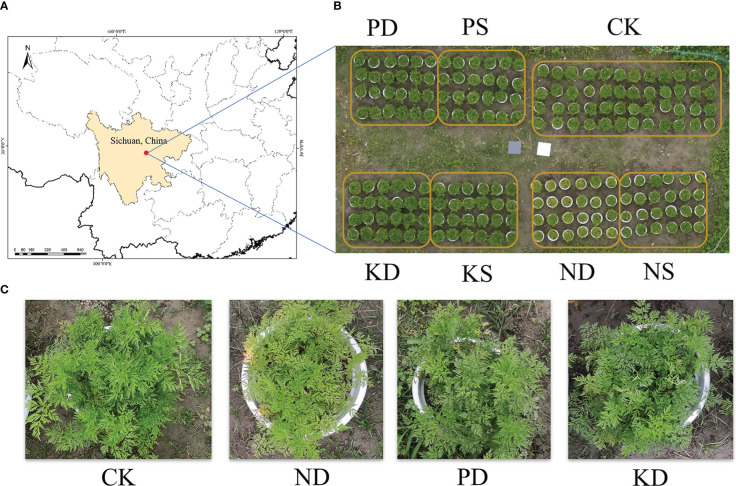
Study site, experimental design, and stress characterization. **(A)** Study area location, **(B)** Study area, **(C)**, Stress characterization. CK, control group, ND, nitrogen deficiency group, PD, phosphorus deficiency group, KD, potassium deficiency group, NS, nitrogen supplementation group, PS, phosphorus supplementation group, KS, potassium supplementation group.

The field experiment was conducted in the Medicinal Botanical Garden of Chengdu University of Traditional Chinese Medicine (30°69’N, 103°81’E, 524m ASL) located in Chengdu City, Sichuan Province, China, from January 2022 to June 2022 (**Figure 2A**). The region has a humid subtropical monsoonal climate. The average temperature during the experiment was 13.7°C, and the accumulated rainfall was 316.99 mm. The cultivation medium is made of yellow loam, perlite, and coconut coir in a volume of 5:3:2. The yellow soil was collected from long-term unfertilized plots (pH 6.98, organic matter content of 18.4 g/kg, available nitrogen content of 43.71 mg/kg, available phosphorus content of 19.57 mg/kg, and available potassium content of 51.92 mg/kg). After the soil was air-dried for several days, it was crushed and passed through a 5 mm sieve (Rajkovich et al., 2011). The mixed cultivation medium was packed into polypropylene pots with quartz sand at the bottom, and 2/3 of the pots were buried in the soil and kept at the same height.

The germplasm material was crop rhizomes harvested from Fengdui Village, Dujiangyan City, Sichuan Province. The area is a Geo-Authentic product area of L. chuanxiong. Before planting, remove the aerial parts and fibrous roots according to traditional planting habits. After 3 days of placement, choose rhizomes of even size for planting. Two in each pot are one sample, totaling 196 samples. Hoagland’s nutrient solution was watered weekly after planting to ensure normal growth in the early stages (Hoagland and Arnon, 1950). Until April 1, samples were divided into control (CK), N deficient (ND), P deficient (PD), and K deficient (KD) groups. Each processed 48 samples. Every 7 days, 500 ml of the corresponding nutrient solution was poured, CK was poured with Hoagland’s nutrient solution, and the stress group was poured with Hoagland’s nutrient solution with the relative mineral elements completely removed. The deficient nutrient solution was prepared according to the method of Xu and Mou (2016). Watering the soil with sufficient water to remove the pre-watering Hoagland’s solution before starting the treatment. After 30 days of treatment, 24 samples were divided from ND, PD, and KD as nitrogen supplementation group (NS), phosphorus supplementation group (PS), and potassium supplementation group (KS), respectively, and changed to watering with whole Hoagland nutrient solution (**Figure 2B**).

#### 2.1.2 Remote sensing image acquisition

A total of nine missions were conducted during the experiment in April-May 2022 to capture multispectral images between 11:00 and 13:00 in cloudless and windless weather. The interval between each capture was about 7 days. The drone used is the DJI Phantom 4 Multispectral (DJI, Shenzhen, China), which was equipped with a multispectral lens having six CMOS sensors, including one RGB sensor for visible imaging and five single-band sensors (B: 450 ± 16 nm, G: 560 ± 16 nm, R: 650 ± 16 nm, RE: 730 ± 16 nm, NIR: 850 ± 26 nm). Missions were uploaded to the drone *via* DJI GS Pro. Above ground level (AGL) was set to 5 and 10 meters. Under this AGL, the drone did not affect the crop canopy, and the orthoimage stitching was normal. The ground sampling distance (GSD) was 0.265 cm/pixel (5m AGL) and 0.529 cm/pixel (10m AGL). The camera was connected to the drone with a gimbal, and shooting angle was 90° from the ground. The for-ward overlap rate was 80%, and the side overlap rate was 75%. Image geographic coordinates determined by Real Time Kinematic (RTK) GPS with an error of less than 1 cm in the horizontal direction and less than 1.5 cm in the vertical direction. The 10% and 90% radiometric calibration plates (JINGYI, Guangzhou, China) were placed in the center of the plot before the mission begin. It was used to verify the radiometric calibration effect.

### 2.2 Digital image processing and data analysis

#### 2.2.1 Generation of orthorectified mosaic and radiometric correction

The generation of orthorectified mosaic was done on DJI Terra (DJI, Shenzhen, China) and the steps include radiometric calibration, image alignment, dark angle compensation, and aberration calibration. The radiometric calibration was calculated as follows (DJ-Innovations, 2020):


(1) $$X_{ref}=\frac{X_{DN}\times pCam_{X}}{X_{LS}\times pLS_{X}}\times \rho_{NIR}$$

Where X is the response band, *X_DN_* is the brightness value of the image element in this band, *X_LS_* is the light-sensitive signal obtained by the light intensity sensor, *ρ_NIR_* is the parameter that regulates the interconversion between the NIR image signal and the multispectral light intensity sensor, and *pCam_X_* and *pLS_X_* are the calibration parameters obtained by the multispectral light intensity sensor in other bands with reference to the NIR band.

#### 2.2.2 Feature extraction and variable screening

**Figure 3:**
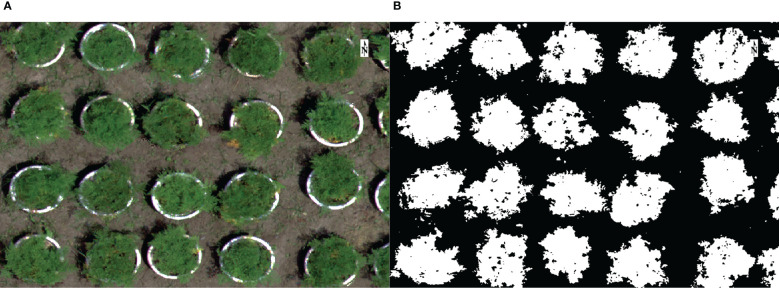
Mask extraction. Support vector machines (SVM) separated the crop crown from the background. **(A)** RGB image. **(B)** Mask.

Mask images were made using the support vector machine (SVM) algorithm (**Figure 3**), and vegetation indices were calculated (**Table 1**). Then the image was segmented, and mask extracted the sample mean reflectance and vegetation index (Hassanzadeh et al., 2020), then removed redundant variables through the information value (IV) and correlation between variables (Zaghwan and Gunawan, 2021). Correlation coefficients between variables were calculated by person correlation analysis, and 90% was used as the correlation threshold to remove redundant variables (Hassanzadeh et al., 2020). The IV is used primarily to evaluate the predictive ability of variables in the classification model. The higher the IV value, the higher the information contribution of the variable. Before calculating IV, the data needs to be discretized. The calculation formula is as follows (Zhang et al., 2017):

**Table 1:** Vegetation indices, equations, and sources used in the study.

VIs	Name	Formula	References
NDVI	Normalized Difference VI	*NDVI*=(*NIR–R*)/(*NIR*+*R*)	(Rousel et al., 1973)
RVI	Red Ratio VI	*RVI*=*NIR*/*R*	(Jordan, 1969)
EVI	Enhanced VI	$EVI=2.5(\frac{NIR-R}{NIR+6R-7.5B+1})$	(Huete et al., 2002)
DVI	Difference VI	*DVI*=*NIR*–*R*	(Richardson et al., 1977)
RDVI	Renormalized Difference VI	$RDVI=(NIR-R)/\sqrt{NIR-R}$	(Roujean and Breon, 1995)
SAVI	Soil Adjusted VI	*SAVI*=1.5(*NIR*–*R*)/(*NIR*+*R*+0.5)	(Huete, 1988)
GNDVI	Green Normalized Difference VI	*GNDVI*=(*NIR*–*G*)/(*NIR*+*G*)	(Gitelson et al., 1996)
NDRE	Normalized Difference Red-edge VI	*NDRE*=(*NIR*–*RE*)/(*NIR*+*RE*)	(Gitelson et al., 1994)
OSAVI	Optimization of Soil-Adjusted VI	*OSAVI*=(*NIR*–*R*)/(*(NIR*+*R*+0.16)	(Rondeaux et al., 1996)
GRVI	Green Ratio VI	*GRVI*=*NIR*/*G*	(Xue et al., 2004)
LCI	Leaf Chlorophyll Index	*LCI*=(*NIR*–*RE*)/(*NIR*–*R*)	(Datt, 1999)
NDWI	Normalized Difference Water Index	*NDWI*=(*G*–*NIR*)/(*G*+*NIR*)	(Gao, 1996)
BNDVI	Blue Normalized Difference VI	*BNDVI*=(*NIR*–*B*)/(*NIR*+*B*)	(Peñuelas et al., 1995)
BVI	Blue Ratio VI	*BVI*=*NIR*/*B*	(Jordan, 1969)
BRVI	Simple Blue Ratio Index	*BRVI*=*R*/*B*	(Peñuelas et al., 1994)


(2) $$IV=\sum_{i}^{n}((y_{i}/y_{T}-n_{i}/nT)\times ln(\frac{y_{i}/n_{i}}{y_{T}/nT}))$$

Where n is the number of groups, set to 10; i represents the ith group; *y_i_* is the number of positive samples in this group; *n_i_* is the number of negative samples in this group; *y_T_* is the number of all positive samples in the sample; *nT* is the number of all negative samples in the sample; to prevent extreme values, if the number of positive samples or negative samples in the variable group is 0, it is adjusted to 1.

#### 2.2.3 Data analysis and evaluation

Data processing and evaluation were performed in Python 3.8. Divide the data into training and test sets according to 7:3. Standardize and PCA dimensionality reduction of selected variables (Abdi and Williams, 2010). Since the dataset is an unbalanced sample, the SMOTE algorithm was used to oversample the training set data (Zhu et al., 2017). And then, the model was trained using K-Nearest Neighbor (KNN), Logistic Regression (LR), Naive Bayesian Model (NBM), Support Vector Machine (SVM), Decision Tree (DT), and Random Forest (RF) algorithms. The optimal parameters of the model were determined by grid search and five-fold cross-validation. Model performance was evaluated by AUC (Area under the Curve), precision, recall, and f1-score. All evaluation metrics were averaged over ten random divisions of the training and test sets obtained (Hossin and Sulaiman, 2015). AUC is the area under the ROC curve, which is applicable to the evaluation of classification models with unbalanced samples. The closer the AUC is to 1, the better the model is; close to 0.5, the model has no predictive value. Precision indicates the proportion of true cases among positive cases, recall indicates the proportion of true cases among all positive cases, f1-score neutralizes the precision and recall for evaluation, and the calculation equation is as follows (Wu et al., 2022):


(3) $$precision=\frac{TP}{TP+FP}$$

(4) $$recall=\frac{TP}{TP+FN}$$

(5) $$F1\_score=\frac{2\times precision\times recall}{precision+recall}$$

Where TP is the number of samples where the instance is a positive class and is predicted to be positive, TN is the number of samples where the instance is a negative class and is predicted to be negative, FN is the number of samples where the instance is a positive class and is predicted to be negative, and FP is the number of samples where the instance is a negative class and is predicted to be positive.

### 2.3 Ground sampling and chemical analysis

Ground sampling activities were conducted before each nutrient watering (16:00-18:00 on the same day), and nine sampling sessions were conducted. SPAD was measured with MultispeQ V2 (PhotosynQ, USA) by selecting the first fully expanded leaf below the terminal branch and measuring the mean of five parts of the leaf on both sides of the base, both sides of the middle, and the tip. Each treatment was randomly sampled 10 times. At the same time, the first fully expanded leaf was collected for chemical analysis of nutrient element content, all samples were collected, and each 8 replicate samples were mixed into 1 sample (about 0.25 g). A minimum of 3 samples per treatment were used for chemical analysis. After collection, they were placed in ice boxes and brought back to the laboratory for chemical assays, washed 2-3 times using RO water dripping, deenzymated at 105°C for 30 min, and dried at 65°C to constant weight. Digest with H_2_SO_4_ -H_2_O_2_, Kjeldahl analyzer (BUCHI K-360, FOSS, Sweden) was used to determine the total K, UV-Vis spectrophotometer (A580, AOE, China) for total P determination, and total K was determined using a flame photometer (6400A, shjingmi, China) (PRC, 2011).

After the last flight mission, dry biomass and leaf-to-stem ratio (LSR) were determined by the weighing method (Smart et al., 2004), and chlorophyll and carotenoid contents in leaves were determined by the acetone extraction colorimetric method (Arnon, 1949).

## 3 Results

### 3.1 Effect of nutritional deficiency on the growth of *L. chuanxiong*

Samples were collected after 58 days of stress and measured for biomass, chlorophyll content, carotenoid content, and leaf-to-stem ratio (**Table 2**). Except for KS, all treatment groups showed a significant decrease in biomass compared to CK, with ND showing the largest decrease of 43.52%, PD and KD decreasing by 21.15% and 14.33%. And biomass increased in all groups after supplementation with deficient nutrients compared to those with complete deficiency. For chlorophyll content, only ND showed significant differences with CK. PD (P=0.121) and KD (P=0.078) showed an increasing trend in chlorophyll content, but there was no significant difference. For carotenoid content, ND was significantly reduced, and PD significantly increased compared to CK. For LSR, all treatment groups showed a decrease compared to CK. The decreases were 52.27%, 42.05% and 18.18% in the KD, ND and PD groups. And LSR increased after supplementation with deficient nutrients compared to the deficient treatment. Collectively, all stress groups caused a reduction in biomass compared to the control group, with ND>PD>KD. Only ND significantly reduced chlorophyll and carotenoid contents. All the stress groups caused a reduction in the leaf-to-stem ratio, where KD>ND>PD.

**Table 2:** Effect of different treatments on plant biomass, chlorophyll content, carotenoid content, and leaf-to-stem ratio.

Group	Dry biomass (g/pot)	Chlorophyll (mg/g)	Carotenoid (mg/g)	Leaf-to-stem ratio
CK	69.75 ± 8.41	0.83 ± 0.15	0.19 ± 0.02	0.88 ± 0.14
ND	39.39 ± 5.25**	0.54 ± 0.15**	0.14 ± 0.02**	0.51 ± 0.09**
NS	48.73 ± 5.53**	0.8 ± 0.18	0.18 ± 0.03	0.66 ± 0.16**
PD	55.002 ± 9.50**	0.91 ± 0.14	0.22 ± 0.03*	0.72 ± 0.05*
PS	57.26 ± 4.97*	0.85 ± 0.2	0.21 ± 0.03	0.71 ± 0.12*
KD	59.75 ± 11.09*	0.92 ± 0.13	0.21 ± 0.03	0.42 ± 0.06**
KS	61.44 ± 10.51	0.85 ± 0.16	0.20 ± 0.03	0.52 ± 0.07**

* (P<0.05) and **(P<0.01) represent significant differences from the control group. Statistical methods used were Student’s t-test.

### 3.2 Model building and evaluation

#### 3.2.1 Variable filtering

**Figure 4:**
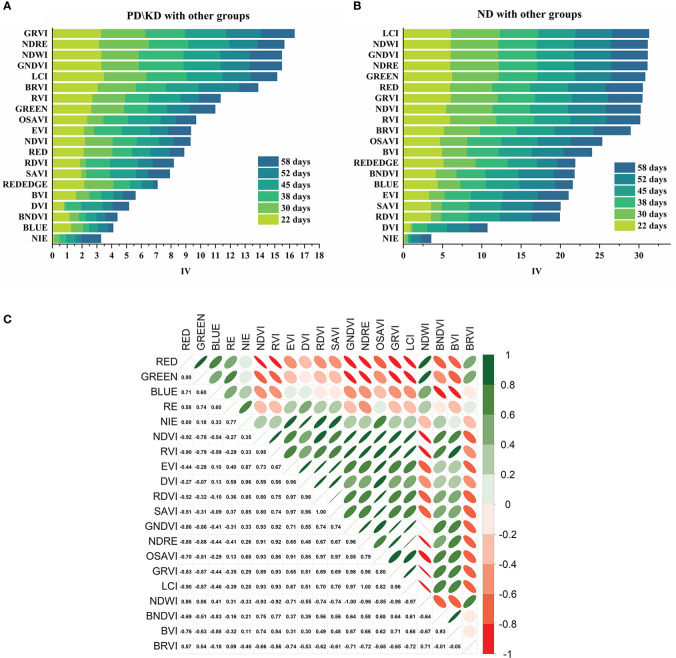
Variable screening based on IV and correlation. **(A)** The IV was calculated with PD\KD as the positive sample and the other treatment groups as the negative sample. **(B)** The IV was calculated with the ND as the positive sample and the other treatment groups as the negative sample. **(C)** Heat map of vegetation index correlation.

Including single-band reflectance and vegetation index, we counted 20 indicators as pre-selected variables (**Figure S1**). To remove redundant information and simplify the workflow by information value (IV) and Pearson correlation analysis. We used IV as the degree of variable contribution and 0.9 as the correlation threshold (**Figure 4**) and finally determined the GRVI, LCI, BRVI, RVI, GREEN band, RED band, RE band, OSAVI, BVI, EVI as the input variable.

**Figure 5:**
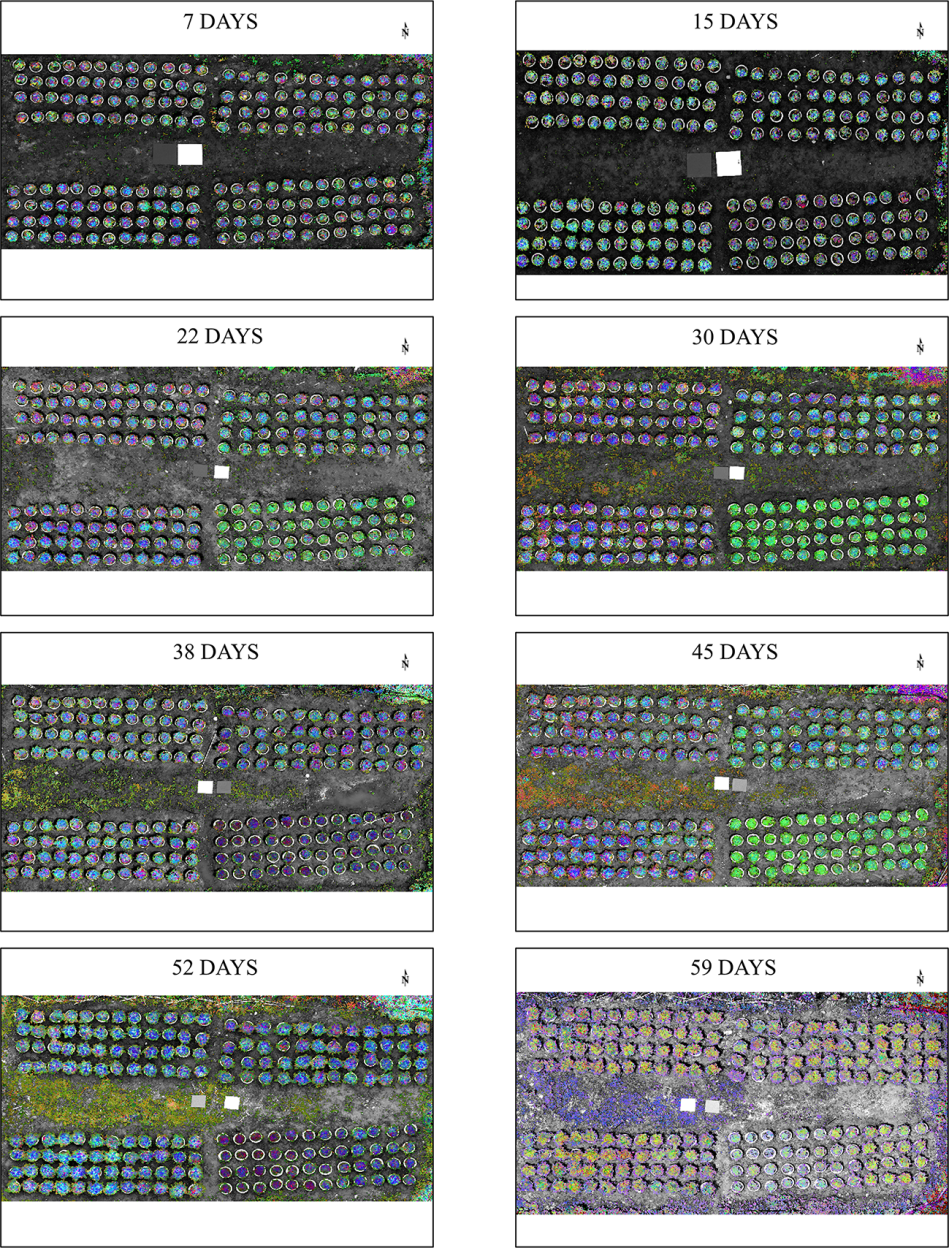
Dimensionality reduction images at different stress times. The remote sensing images of selected variables were subjected to PCA downscaling. b: PC1, g: PC2, r: PC3.

We removed the background of the selected variables and conducted PCA dimension reduction. As shown in **Figure 5**, with increasing stress time, phenotypic changes were first seen in the ND group (After 15 days). After 22 days of stress, the PD and KD groups began to show differences from the CK group. After 30 days of stress, we set up a supplemental fertilizer treatment, and the supplemental fertilizer treatment group gradually returned to the level of the CK group.

#### 3.2.2 Different algorithms and AGL evaluation

**Figure 6:**
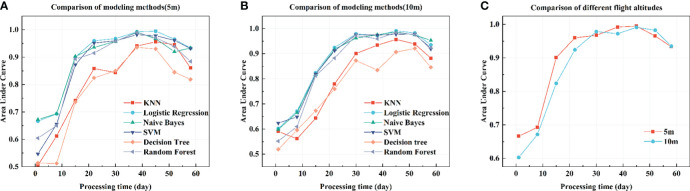
Different modeling algorithms and AGL evaluation. AUC is the evaluation metric of the model as the average of area of the ROC curves for each classification sample. **(A)** performance evaluation of different algorithms at 5 m AGL, **(B)** evaluation of different algorithms at 10 m AGL, **(C)** evaluation of logistic regression algorithms at different AGL.

The classification effects of different algorithms under 5 m and 10 m AGL were compared (**Figures 6A, B**), with AUC as the evaluation criterion. LR maintains the optimal classification performance under different stress stages and heights; NBM, SVM, and RT also have high classification performance, while Decision Tree and KNN perform poorly. After 23 days of stress, the AUC values of the models constructed by LR, NBM, SVM, and RT algorithms reached or approached 0.9. The classification effect was the best at 38 and 45 days of stress, and the AUC values of the LR models exceeded 0.99. After 52 days, the classification performance of all algorithms and flight altitude models decreased. Overall, LR is best for building models.

Comparison of the LR algorithm at different heights (**Figure 6C**). The classification effect of 5m AGL was higher than that of 10m AGL before 22 days of stress. The AUC reached above 0.9 after 15 days of stress, while the AUC exceeded 0.9 after 22 days with 10 m AGL. Both models had similar classification performance after 31 days. AUC all reached above 0.99 after 45 days of stress. Overall, modeling efficacy was similar for 5m and 10m collection data after 30 days of stress, but 5m AGL modeling was more sensitive to nutrient deficiency.

#### 3.2.3 Model evaluation

**Figure 7:**
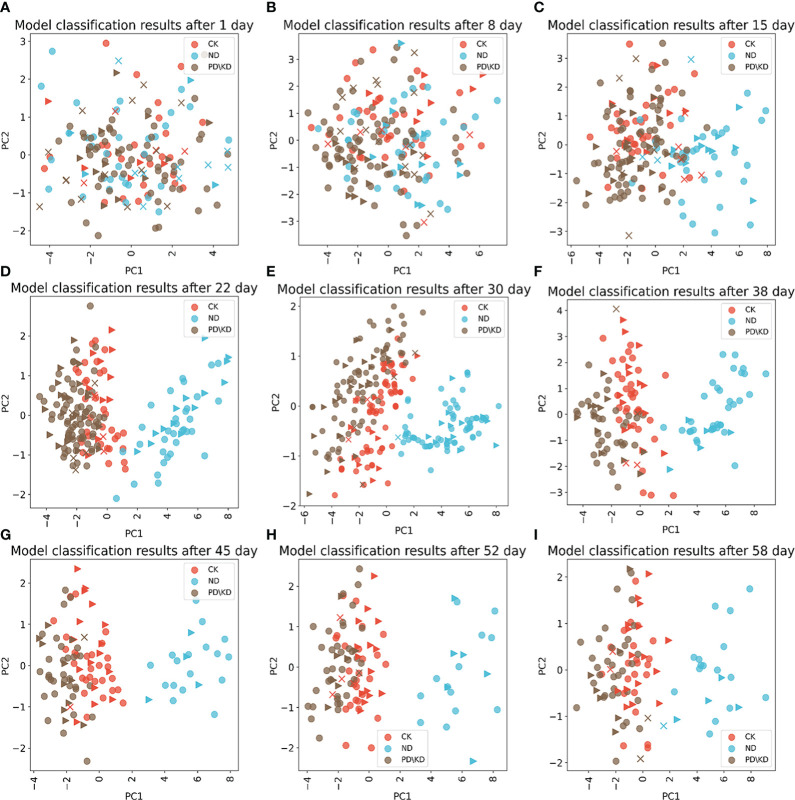
Classification results of logistic regression(LR) models under different stress time models. PCA dimensionality reduction and visualization of sample data collected at 5 m height. **(A-I)** were the classification results after 1, 8, 15, 22, 30, 38, 45, 52 and 58 days of stress successively. The LR algorithm predicts the test samples. ▼ are the correctly predicted samples in the test set, × are the incorrectly predicted samples in the test set, and • are the training set samples.

Based on the results in the above sections, we choose to use the LR algorithm to build the model under 5m AGL and perform PCA dimensionality reduction visualization for samples in different stress periods (**Figure 7**). After 15 days of stress, the ND group was gradually separate from the CK and PD\KD groups (**Figure 7C**), and the prediction accuracy was 78.48%. And the accuracy rate reached 97.77% after 22 days. From 38 to 58 days, the prediction precision and recall rate both reached 100% (**Table 3**). After 22 days of stress, there were differences between PD\KD group and CK group (**Figure 7D**), the recognition precision rate reached 87.1%, and the recall rate was 82.08%. The recognition accuracy rate between 30 and 45 days was between 87.3% and 92.35%, the recall rate was between 86.37% and 89.03%, and the recognition effect was the best (**Table 3**). The recognition rate decreased in both ND and PD\KD groups after 52-58 days of stress.

**Table 3:** Evaluation index of models in different stress stages.

Processing time(day)	CK			ND			PD\KD		
	precision	recall	f1-score	precision	recall	f1-score	precision	recall	f1-score
1	0.4834	0.46178	0.46028	0.37892	0.46428	0.4129	0.5881	0.51908	0.55046
8	0.31558	0.30658	0.3061	0.45906	0.5562	0.50048	0.57894	0.51332	0.54098
15	0.3868	0.4857	0.42704	0.7848	0.72878	0.7511	0.68514	0.63372	0.65652
22	0.66568	0.7395	0.69776	0.97772	0.98824	0.98284	0.871	0.8208	0.8439
30	0.71898	0.82944	0.76458	0.98888	0.92174	0.9526	0.92354	0.86366	0.88972
38	0.91042	0.90094	0.90274	1	1	1	0.87308	0.8903	0.87636
45	0.89516	0.91098	0.90098	1	1	1	0.91516	0.87378	0.89086
52	0.91714	0.83096	0.8659	1	1	1	0.83254	0.9159	0.86614
58	0.77534	0.8472	0.80246	1	0.89642	0.93846	0.85016	0.81076	0.82456

### 3.3 Comparison with other diagnostic methods and field validation

**Figure 8:**
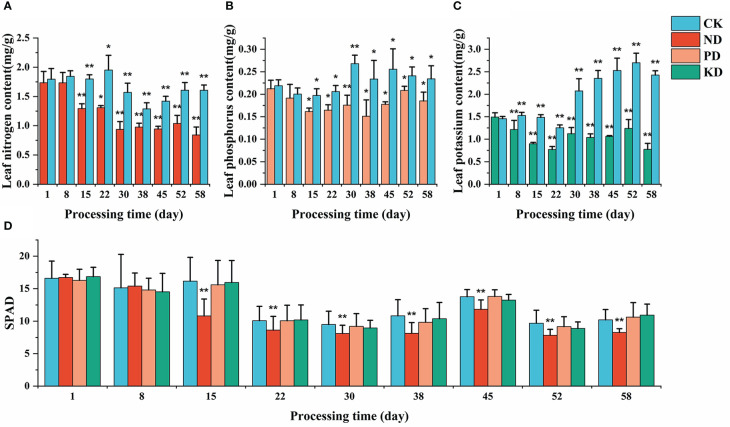
Chemical diagnosis and SPAD diagnosis results. **(A)** Leaf N content. **(B)**: leaf P content. **(C)** leaf K content. **(D)** SPAD values; * (P<0.05) and ** (P<0.01) represent significant differences from the control group. Statistical methods used were Student’s t-test and ANOVA.

Diagnosis of each treatment group was performed by chemically measuring the elemental content of the plant leaves and SPAD (**Figure 8**). There was a significant difference compared to the CK group, indicating that the diagnostic method could make a valid diagnosis of stress in that period. For the ND group, both chemical diagnosis and SPAD diagnosis showed significant differences from the CK group after 15 days of stress (**Figures 8A, D**); After 15 days of stress, the images of RGB, GRVI, and results of PCA were different from those of CK group (**Figures 5**, **7C**). For the P deficiency treatment, leaf P content was significantly different between the CK group after 15 days (**Figure 8B**), while there was no difference in SPAD compared to the CK group. There was a difference between PCA images after 22 days of stress (**Figure 5**), which was further proved by PCA scatter plot (**Figure 7**). For the K deficiency treatment, leaf K content was significantly reduced after 8 days of stress compared to the CK group, and there was no significant difference in SPAD. The diagnosis period of potassium-deficient plants by multispectral imaging was 15 to 22 days after stress. Based on this, multispectral diagnosis is similar to chemical diagnosis in the diagnosis period of nitrogen deficiency, while phosphorus and potassium deficiency are slightly lagged behind.

**Figure 9:**
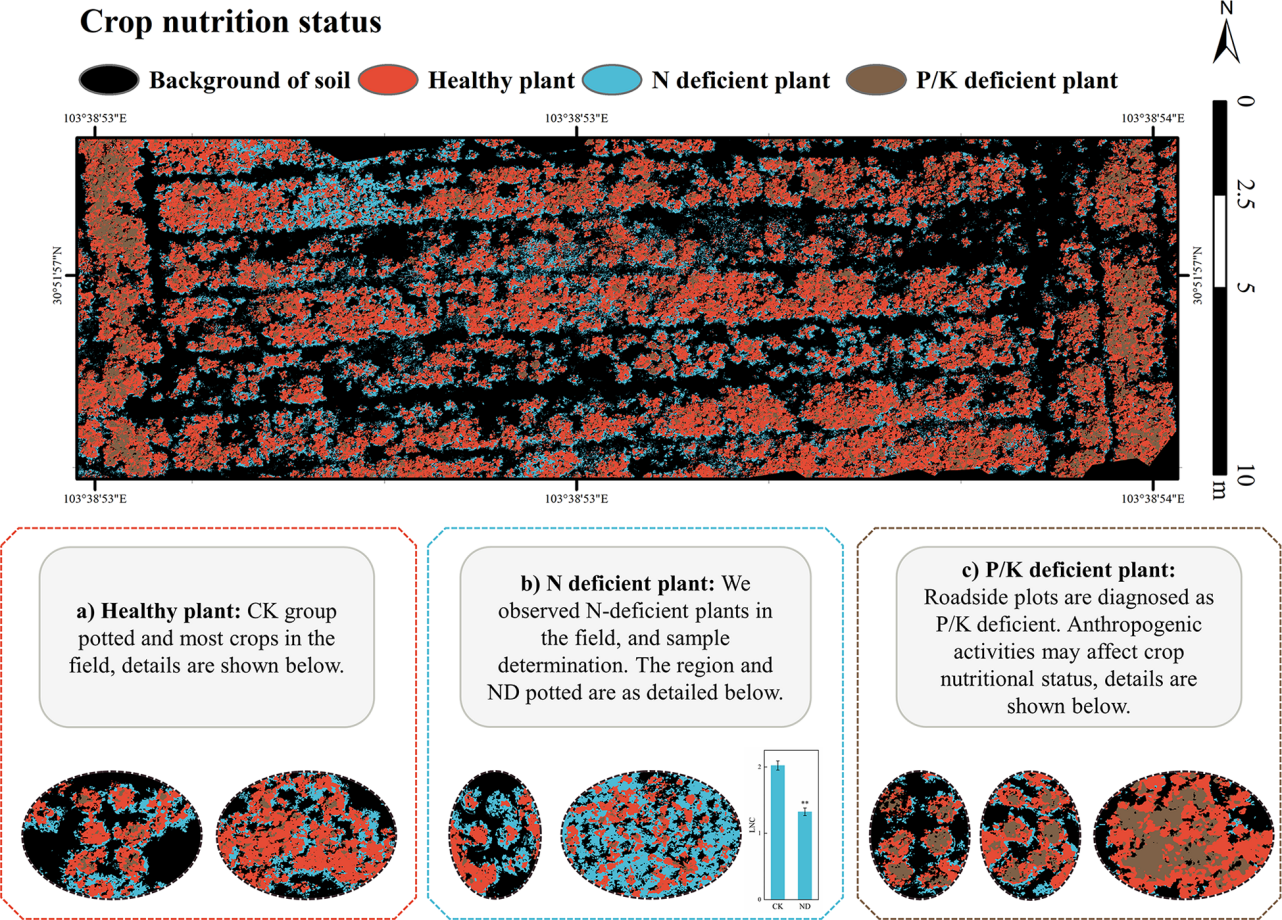
Field verification (5m). **(P<0.01) represent significant differences from the control group. Statistical methods used were Student’s t-test.

As shown in **Figure 9**, we predicted the nutrient status of the field vegetation in *L. Chuanxiong* planted fields with the model developed during the same period (after 38 days of stress). In this image acquisition, potted plants of ND, PD, and KD groups were placed in the open area of the field. We collected leaves from N deficient area and normal field, and N content of the leaves in this area was significantly lower than that of normal field leaves. Moreover, the results predicted by the model were similar to the ND group of crops (**Figure 9B**). Crops in most areas and the CK group were predicted to be healthy vegetation (**Figure 9A**). Crops in the roadside area were predicted to be phosphorus or potassium deficient, similar to the results predicted for the PD and KD groups (**Figure 9C**).

## 4 Discussion

### 4.1 Effect of N deficiency on crop phenotype and canopy spectrum

N is an essential nutrient for plants’ main physiological metabolic functions and is closely related to chlorophyll synthesis and light metabolism. Under our experimental conditions, nitrogen deficiency produced distinct symptomatic features with uniform yellow leaves and slow plant growth (**Figure 2C**). Yellowing symptoms occurred first in the basal leaves and later caused the yellowing of the whole plant.

In agreement with Have et al. (2017), N deficiency caused a decrease in chlorophyll and carotenoid content in the leaves (**Table 2**), while a decrease in the pigment content of canopy leaves followed by an increase in visible light reflectance was the key to identifying N-deficient plants. N deficiency caused slow crop growth and a significant reduction in LSR (**Table 2**), which resulted in sparse vegetation canopy foliage. Although background segmentation was performed prior to data processing, mixed image elements still resulted in spectral differences (Benincasa et al., 2017), which is also an important factor in identifying N-deficient plants. Therefore, the key to distinguishing N-deficient plants is the canopy pigment content and the number of canopy leaves.

The indices LCI, NDWI, GNDVI, and NDRE in our study contributed more information gain to the identification of N-deficient plants than single bands (**Figure 4A**). This is consistent with the finding of Osco et al. (2020) that vegetation indices contributed more to the prediction of leaf N content than spectral bands. Meanwhile, the green and red band reflectance provided a high information gain (**Figure 4A**) and a weak correlation with the vegetation index (**Figure 4C**), which is also consistent with the finding of Li et al. (2022) that the combination of vegetation index plus spectral band variables can improve the accuracy of the model. In addition, we verified by supplementing the treatments with deficient elements that the change was indeed due to differences in N deficiency. All indices and bands except the red-edge band tended to move closer to the control after the N supplementation treatment (**Figure 5**, **Figure S1**), with indices such as OSAVI and RVI being more sensitive to the response of N supplementation.

### 4.2 Effect of P and K deficiencies on crop phenotype and spectrum

Plants are usually subjected to P deficiency conditions, where the reduction in cell division and elongation leads to high chlorophyll concentration and further causes anthocyanin accumulation, giving the leaves a purplish-red color (de Bang et al., 2021). However, Hughes and Lev-Yadun (2015) found that reddening leaf margins were not a common symptom of all P deficiencies. For example, in sugar beet, rice, and potato, P deficiency symptoms only manifested as stunted growth with dark blue/green leaf coloration. Under our experimental conditions, only a few plants were observed to have reddish-purple leaves in the early stages of stress, but the leaves were dark green with little new leaf emergence (**Figure 1C**).

The present study differs from Gracia-Romero et al. (2017)’s study in that P deficiency increased NDVI, GNDVI, LCI, and other indices (**Figure S1**). The difference could be the accumulation of more chlorophyll under P-deficient conditions and the reduction in the number of new leaf sprouts in the canopy or the difference in the GSD, making the previous spectral images contain more information about the soil background.

Under K-deficient conditions, plants generally exhibit symptoms of chlorosis or necrosis from the tip to the edge of old leaves (Ueno et al., 2018) and loose leaves and stems. In this experiment, the symptoms of edge necrosis of old leaves were not easily detected, but the plants showed obvious relaxation of leaves and stems (**Figure 1C**). At the same time, the number of new leaf germinations was significantly reduced compared with normal plants.

K deficiency greatly reduced the leaf-to-stem ratio of crops (**Table 2**), indicating that K deficiency limited the reduction of crop new leaf germination, and the reflectance of new leaves in the visible light band is lower than that of mature leaves (Nakaji et al., 2019; Wu et al., 2022). The reduction of the visible light band in the canopy of K-deficient plants was related to the decrease in the proportion of young leaves in the canopy caused by K deficiency. This spectral change is similar to that of Severtson et al. (2016) for diagnosing K deficiency in rapeseed by a drone-carrying canopy sensor. Unlike the study of Furlanetto et al. (2021), the former study found that the chlorophyll concentration, GNDVI, RVI, and GRVI of maize decreased in severe K deficiency. In this study, the chlorophyll concentration of crops did not decrease under the state of P deficiency but increased compared with normal plants; the GNDVI, RVI, GRVI, and other indices were significantly higher than normal plants. This may be related to the reduction of the new leaf germination of *L. chuanxiong* and the higher spatial resolution in this study. It is worth noting that GRVI obtained the best regression model between K content in the former study and the maize growth stage, and in this study, GRVI was also the best index to distinguish PD\KD groups from other groups (**Figure 2B**).

In the model constructed in this study, PD and KD groups were set as one category because the canopy of *L. chuanxiong* under P and K deficiency treatments had similar spectral characteristics and phenotypic changes. However, compared with potassium deficiency, phosphorus deficiency did not severely limit the germination of new leaves. The reason for the spectral change may be the dark green overall appearance of the plant due to the accumulation of pigment. Increasing the band of the multispectral camera or adding texture information may be the solution. In practice, this method should be applied for initial diagnosis in large-scale production and combined with other means to further determine phosphorus or potassium deficiency.

### 4.3 Effects of GSD and classifiers on model performance

Background information, such as exposed soil and vegetation shading, may significantly impact the vegetation index, especially in the case of small canopy coverage (Benincasa et al., 2017). Removing the background does not always improve the results, and the solution to the problem is usually to increase the image’s resolution (Corti et al., 2018). In the present study, lower AGL improved the model’s accuracy at an early stage (15-22 days of stress). However, the higher recognition accuracy (1-8 days of stress) before differences in chemical assays led us to consider that lower AGL are more susceptible to noise. While the model constructed with 10 m AGL had lower classification performance in the early stage, it achieved similar classification performance after 30 days of stress (**Figure 6C**). There was also no significant change in accuracy when Vega et al. (2015) used multispectral images to monitor sunflower nitrogen status with GSDs ranging from 1 to 100 cm/pixel. We argued that different GSD does not affect the accuracy of model recognition, and using a lower AGL only means increasing the model sensitivity at the early stage of stress, but it may also reduce the model noise resistance.

We compared the model performance of KNN, LR, NBM, SVM, DT, and RF with AUC as the evaluation index and found that LR, NBM, SVM, and RF all achieved better prediction accuracy at 5m and 10m AGL. Among them, the LR algorithm achieved the best results in each stress period and flight altitude, but the model sensitivity was high and easily affected by noise at 5m AGL. Both NBM and RT have such problems, while SVM performs better on this problem. The study by Zermas et al. (2015) also showed that the LR algorithm showed higher sensitivity than the SVM algorithm in distinguishing N-defective leaves. KNN performs classification by measuring the distance method between different feature values. NBM is a probabilistic classification method proposed by Pearl based on Bayes’ theorem. DT judges the attributes of samples sequentially based on knowing the probability of occurrence of various situations until the final result is derived. RF is an integrated algorithm consisting of multiple decision trees. SVM and LR are classification methods based on linear models, and the results of the two algorithms are very close in most experiments. The SVM is a structural risk minimization model, which is not easily affected by outliers. This is why SVM was not affected by noise in this study, but it also means that it is not sensitive to vegetation diagnosis at the initial stage of stress. Classifiers based on linear discriminant always achieve better classification results in the classification of remote sensing images, such as SVM and LDA (Ang and Seng, 2021; Zhang et al., 2021), and the same is true in this study. All classifiers showed a decrease in performance at the later stage of stress, which is related to plant physiological characteristics.

### 4.4 Consistency inspection with traditional diagnostic methods and field validation

In previous studies, vegetation indices such as NDVI, GNDVI, and NDRE showed high sensitivity to leaf nitrogen content (Gordillo-Salinas et al., 2021; Rehman et al., 2022), while P and K deficiency treatments only responded to severe deficits (Gracia-Romero et al., 2017; Furlanetto et al., 2021). The present study’s spectral responses of P and K deficiency treatments also showed delayed diagnosis time. We diagnosed K deficiency symptoms after 7 days of stress and P and N deficiency symptoms after 15 days of stress by chemical assays (**Figure 8**). The spectral response of the ND group appeared at the same time as the difference in leaf N content. The spectral response of the PD and KD groups appeared 7 to 14 days after the appearance of the elemental difference (after 22 days of stress). It can be seen that UAV multispectral technology lags behind the chemical diagnosis of P and K deficiency symptoms but can diagnose N-deficient plants promptly. Although the remote sensing image diagnosis method in this study is relatively slow in the diagnosis of phosphorus and potassium deficiency, it is more suitable for large-scale agricultural production than the chemical diagnosis method, which needs to rely on a laboratory environment and complex operation.

We verified the feasibility of the practical application of the model in field. Unfortunately, areas predicted by the model to be phosphorus or potassium deficient were not sampled, resulting in our inability to rule out whether the crop in that area was affected by other factors that influenced the results. However, the model successfully identified crops of ND, PD, and KD groups in fields. We cannot strictly control soil nutrient conditions in the field, so constructing an accurate remote sensing nutrient deficiency diagnostic model is difficult. The method used in the paper can provide a solution bill for this purpose, but it is difficult to achieve large-scale cultivation, so it needs to be collected at a lower flight altitude. However, higher flight altitude means higher efficiency, so the question of how models built at lower flight altitudes can be applied at higher flight altitudes will be a further research direction.

## 5 Conclusions

In conclusion, this study developed a nutrient deficit recognition technology based on UAV multispectral images in *L. Chuanxiong* and completed the process from nutrient deficiency model construction to field application. Moreover, we evaluated the influence of different algorithms and flight altitude on the recognition model during the full growth period. It can provide a reference for the application of UAV remote sensing technology in intelligent agriculture and help *L. Chuanxiong* cultivation personnel and botanists to make decisions. In addition, with the rapid development of UAV remote sensing technology, UAV with different sensors will play a greater role in the development and utilization of medicinal plant resources and regulate the production methods of medicinal plant resources in a more reasonable and efficient way.

## Data availability statement

The original contributions presented in the study are included in the article/**Supplementary Material**. Further inquiries can be directed to the corresponding author.

## Author contributions

WL and ZY contributed the central idea, analysed most of the data, and wrote the initial draft of the paper. The remaining authors contributed to refining the ideas, carrying out additional analyses and finalizing this paper. All authors contributed to the article and approved the submitted version.
